# The relationship of aging, complete tooth loss, and having a dental visit in the last 12 months

**DOI:** 10.1002/cre2.309

**Published:** 2020-07-31

**Authors:** Andriana M. Foiles Sifuentes, Maira A. Castaneda‐Avila, Kate L. Lapane

**Affiliations:** ^1^ Department of Population and Quantitative Health Sciences University of Massachusetts Medical School Worcester Massachusetts USA

**Keywords:** aging, edentulous, health services research

## Abstract

**Objectives:**

To evaluate the extent to which dental health care visits in the past year differed among older adults with and without edentulism.

**Material and Methods:**

We conducted a cross‐sectional study using the 2017 Medical Expenditure Panel Survey among participants aged ≥50 years (n = 10,480, weighted = 112,116,641). Two self‐reported outcome variables were used: loss of all teeth from upper and lower jaws (yes/no) and dental visit in the last 12 months (yes/no). Logistic models were used to estimate adjusted odds ratios (aOR) and 95% confidence intervals (CI).

**Results:**

Overall, 11.4% of the non‐institutionalized U.S. population aged ≥50 years were edentulous; the prevalence was higher in those with advanced age. Adherence to annual oral health visits was 16% among those with edentulism, 52% among those without. The prevalence of dental care visits in the past year was higher among those with advanced age without edentulism, but for those with edentulism, the odds of visiting a dental care provider was lower in all age groups compared to those 50–59 years ((60–69 years): aOR: 0.58, CI:0.36–0.95; (70–79 years): aOR: 0.51, CI: 0.30–0.88; (≥ 80 years): aOR: 0.45, CI: 0.26–0.80)).

**Conclusion:**

Although the prevalence of edentulism was higher in those with advanced age, oral health visits during the last 12 months were less frequent in older adults with edentulism. Interventions to improve adherence to dental care recommendations in the growing aging population are warranted.

## 1 INTRODUCTION

As the population ages across the globe, understanding the connection between aging and oral health has become more pressing (Petersen & Yamamoto, 2005). Based on U.S. Census 2018 data, there are ~115 million people aged ≥50 years, with the number expected to increase in coming years (US Census Bureau: American Fact Finder, n.d.). Oral health is intimately linked to aging as biological, behavioral, and socio‐economic factors intersect and interact contributing to declining oral health (AlRahabi, 2019; Freitas et al., 2019; Griffin, Jones, Brunson, Griffin, & Bailey, 2012; Kanasi, Ayilavarapu, & Jones, 2016). Older people lose their teeth; yet age alone is not the sole predictor of tooth loss.

Persons from lower socioeconomic backgrounds and members of vulnerable communities are at a higher risk of tooth loss, oral disease, and edentulism (defined herein as complete tooth loss; Andrade et al., 2019; Bassim et al., 2020; Hybels et al., 2016; Petersen & Yamamoto, 2005; Shelley, Russell, Parikh, & Fahs, 2011). Lack of access to services contributes to oral health disease. Vulnerable communities are often unable to access oral health care providers due to regionality, availability of services, or economic conditions (Doescher, Mouradian, & Brunson, 2010; Griffin et al., 2012; Yoon, Jang, Chio, & Kim, 2018). Treatment for edentulism involves the provision of complete dentures to improve chewing and quality of life (Kroll et al., 2018; Krunic, Kostic, Petrovic, & Igic, 2015; Muller, Morais, & Feine, 2008). Annual visits with oral health care providers are recommended for persons with dental prostheses to evaluate condition and fit (American College of Prosthodontists: Position Statement, n.d.). Recent, population‐based U.S. studies estimating adherence to the guidelines for annual evaluation of prostheses are lacking. Although historically dental insurance among retired people is low (Willink, Schoen, & Davis, 2017), increased enrollment in Medicare Advantage plans offering supplemental dental benefits has improved coverage; four in 10 Medicare Advantage enrollees had dental coverage in 2016 (Willink, 2019).

While supplemental dental insurance included in Medicare Advantage plans may have improved adherence to annual oral health care visits, contemporary studies documenting routine care by age and edentulism status are lacking.

## 2 AIMS

Using a U.S. population‐based data resource, our study sought to provide contemporary estimates of the relationship between age and edentulism among older adults and to evaluate the extent to which dental health care visits in the past year differed among older adults with and without edentulism. We hypothesized that adherence to annual dental health care visits would decline with advanced age and would be greater among people with edentulism relative to those without edentulism.

## 3 METHODS

### 3.1 Ethics statement

Data were collected through a national survey that was approved by the Westat Institutional Review Board and the Office for Protection from Research Risk (Hill, Zuvekas, & Zodet, 2011). Participants provided informed consent. The data were de‐identified and anonymized. Data were released as open‐source and available for public use and pose no risk to participants or individuals collecting the data.

### 3.2 Data source

Data were drawn from the 2017 Medical Expenditure Panel Survey (MEPS), a nationally representative sample of non‐institutionalized U.S. civilians. The Center for Disease Control and the Agency for Healthcare Research and Quality sponsored the data collection for MEPS 2017. Questionnaires were administered to randomly selected persons for household reporting (Hill et al., 2011).

### 3.3 Study population

The MEPS 2017 household component included data from 31,880 participants. We excluded 21,400 participants <50 years of age and responses coded as “refused,” “do not know,” “not ascertained” on complete tooth loss of upper and lower jaws, born in the United States, education, and marital status. The final analytic sample included 10,480 respondents ≥50 years of age (weighted n = 112,116,641). Weighted respondents were cross‐checked with U.S. census estimates for 2017, which totaled 114,217,553 (US Census Bureau: American Fact Finder, n.d.).

### 3.4 Study outcomes

Teeth are fundamental, and pivotal in all aspects of individual and social function including the capacity to macerate food and quality of life (Tan, Peres, & Peres, 2016). As such, our primary study outcome was self‐reported complete tooth loss of all upper and lower teeth (yes/no). The outcome was based on MEPS question: “Have you… lost all upper and lower teeth?” (Griffin et al., 2014).

It is imperative for edentulous persons to maintain an active relationship with an oral health care provider to ensure their prostheses, should they have them, are functioning optimally (American College of Prosthodontists: Position Statement, n.d.). As such, the American College of Prosthodontists recommends annual visits with oral health care providers (Felton et al., 2011). The outcome for the second aim was self‐reported visit with an oral health care provider during 12 months prior to interview. Our outcome was based on MEPS question: “How many dental visits in the last 12 months?” (zero/one or more; Griffin et al., 2014; Meyerhoefer, Zukekas, Farkhad, Moeller, & Manski, 2019).

### 3.5 Covariates

Individual and social characteristics were considered that may influence the ability to access dental services, be it through insurance or financial capacity. Personal characteristics included race/ethnicity, gender, education status (no degree, high school diploma/general education diploma, some college or beyond), born in the United States (yes, no), marital status (married, single, never married), family income as a percentage of the poverty line (poor/negative, near poor, low income, middle income, high income), dental insurance (yes/no), health insurance (private/public/none), dental visit in the last year (yes/no), active smoker in the last 12 months (yes/no). Education status was consolidated into three categories: no degree, high school diploma/general education diploma, and some college or greater. We categorized participants according to their race and ethnicity as Hispanic, non‐Hispanic Black, non‐Hispanic Asian, or non‐Hispanic White. Mixed race/ethnicity persons were included as Hispanic if they identified as such (e.g., Asian‐Hispanic, Black‐Hispanic, White‐Hispanic) or non‐Hispanic mixed race.

### 3.6 Data analysis

**FIGURE 1:**
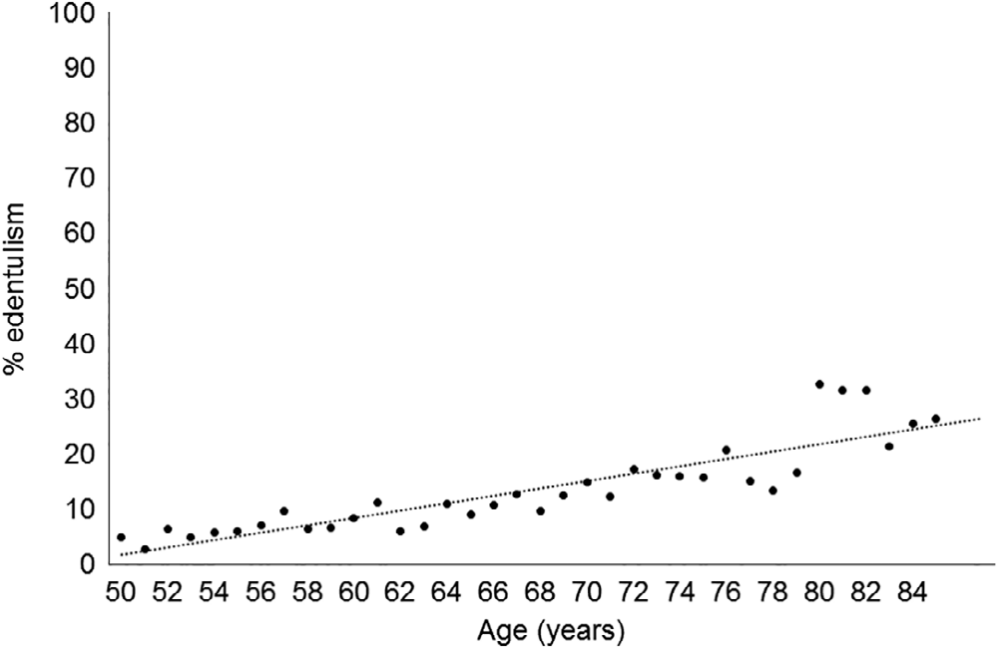
Prevalence of edentulism by age among adults ≥50 years in the United States (2017)

MEPS provided survey weights and approaches for handling single unit datapoints in the weighted measurement were followed (Wun, Ezzati‐Rice, Diaz‐Tena, & Greenblatt, 2007). Descriptive statistics were used to characterize the population according to edentulism. Analyses were stratified by age group. We calculated percentages for categorical variables. Bivariate associations were examined using Pearson Chi square tests for categorical variables. *p*‐Values <.05 were considered statistically significant (two‐sided tests). We then estimated the prevalence of edentulism by age (in years) and depicted this graphically (Figure 1). Logistic regression modeling was used to analyze the relationship between the primary determinant (four categories of age) and edentulism adjusting for potential confounders. We adjusted the partial odds ratio for sex, race/ethnicity, and marital status. We further adjusted the odds ratio for income level, smoking status, and dental insurance.

**FIGURE 2:**
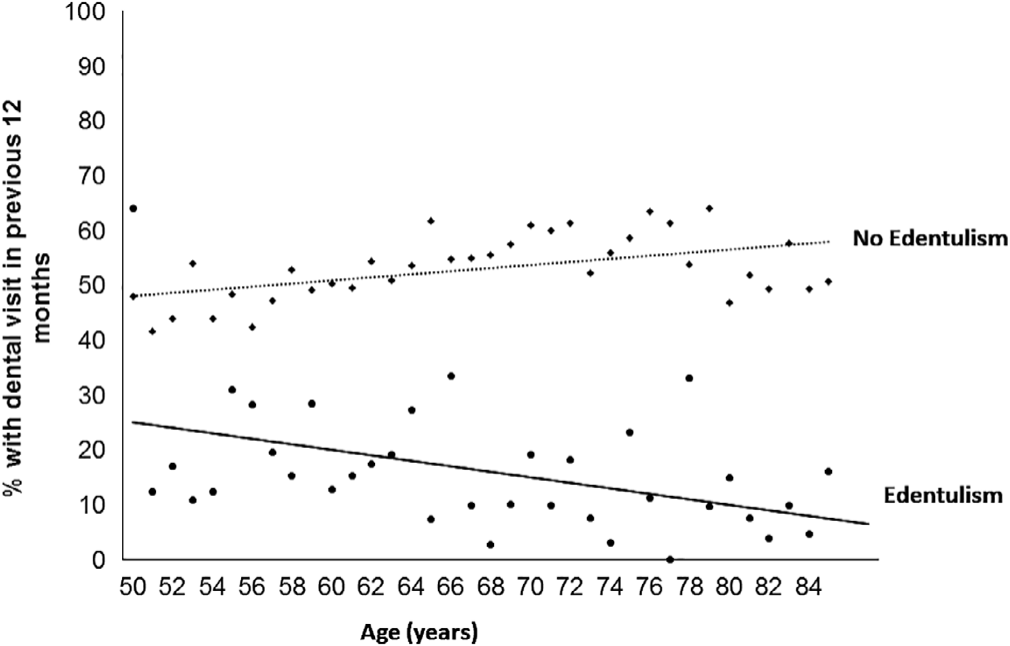
Prevalence of dental visit in previous 12 months by age among adults aged ≥50 years, by edentulism status (2017)

For the second aim, we first estimated the percent of participants who reported having an oral health care visit in the past 12 months, stratified by edentulism status and specific for each year of age (Figure 2). We then conducted a stratified analysis by edentulism status using logistic regression modeling to examine the association between age and visiting an oral health care provider in the last 12 months. Partially adjusted odds ratios included sex, race/ethnicity, and marital status, and the fully adjusted model added family income and dental insurance. Smoking status was excluded from modeling since smoking status has lesser impact on edentulous persons visiting a dental care provider than other potential variables (Dolan, Gilbert, Duncan, & Foerster, 2008; Mittchell & Bennett, 2013). We used STATA version 15.1 (College Station, TX) for all analyses.

## 4 RESULTS

Data from MEPS 2017 indicate that 11.4% of U.S. persons aged ≥50 years of age were edentulous (Table 1), and the prevalence of edentulism increased with age (Figure 1). Table 1 shows the characteristics of adults ≥50 years in the United States, by edentulism status. Overall, about 53% of the population were women and the majority were non‐Hispanic White, which did not vary by edentulism status. While 62.0% of those without edentulism were married, 46.5% of those with edentulism were married. Educational attainment differed by edentulism status with 27.5% of those with edentulism reporting no high school degree or GED compared to 8.9% among those without edentulism. While 51% of those without edentulism reported high income, 24.7% of those with edentulism reported high income. Private insurance was more commonly reported by those without edentulism (70.6 vs. 42.8% among those with edentulism). Public health insurance was twice as common in those with edentulism (53.8%) compared to those that did not have edentulism (25.2%). Dental insurance (edentulism: 15.3% vs. no edentulism: 39.5%) and dental visits in the past year (edentulism: 15.7% vs. no edentulism: 52.2%) differed by edentulism status.

**TABLE 1:** Characteristics of Medical Expenditure Survey Panel (MEPS) participants aged ≥50 years by edentulism (2017)

	Edentulism	
	Yes	No
*Weighted n*	12,758,419	99,354,222
	*Percentage*	
*Women*	53.0	53.3
*Race/ethnicity*:		
Non‐Hispanic Asian	3.5	4.7
Non‐Hispanic Black	12.3	10.3
Hispanic	8.1	11.3
Non‐Hispanic Mixed Race	3.2	2.3
Non‐Hispanic White	72.9	71.4
*Marital status*:		
Married	46.5	62.0
Divorced, widowed, separated	46.7	30.4
Never married	6.8	7.6
*Education*:		
No degree	27.5	8.9
High school diploma	54.1	46.1
Some college or beyond	18.4	45.0
*Born in United States*	88.0	84.0
*Family income as % of poverty line*:		
Poor/negative	17.5	8.3
Near poor	7.0	3.8
Low income	22.0	10.8
Middle income	28.8	26.1
High income	24.7	51.0
*Insurance coverage*:		
Private	42.8	70.6
Public	53.8	25.2
Uninsured	3.5	4.3
*Dental insurance*	15.3	39.5
*Dental visit in the last 12 months*	15.7	52.2
*Active smoker within past 12 months*	16.5	7.0

Table 2 shows the characteristics of older adults in the United States by age group and edentulism status. Across all age groups, fewer people with edentulism were married compared to those without edentulism. For example, among those aged 50–59 years, 51.3% of those with edentulism were married versus 65.4% among those without edentulism. Differences in the distribution of socioeconomic indicators such as educational attainment, income, and health insurance varied between those with and without edentulism, regardless of age group. Those with edentulism were less likely to have had at least some college, were less likely to have high income, and were more likely to have public insurance relative to those without edentulism. For example, among those 50–59 years of age, 17.3% of those with edentulism and 46.0% of those without edentulism had at least some college; 23.1% with and 56.2% without edentulism had dental insurance. Similar patterns were observed across all age groups.

**TABLE 2:** Characteristics of Medical Expenditure Survey Panel (MEPS) participants aged ≥50 years by edentulism, stratified by age (2017)

Age group (years)	50–59		60–69		70–79		≥80	
Edentulism	Yes	No	Yes	No	Yes	No	Yes	No
*Weighted N*	2,589,045	39,509,252	3,563,872	32,951,215	3,427,889	18,602,245	3,176,608	8,303,726
*Women*	51.6	51.2	51.8	53.4	47.3	55.5	61.5	58.3
*Race/ethnicity*:								
Non‐Hispanic Asian	1.9	5.1	4.3	4.7	2.7	4.3	4.8	4.0
Non‐Hispanic Black	12.0	11.5	15.1	10.8	11.7	8.2	10.2	6.8
Hispanic	8.1	14.4	6.4	10.6	8.7	7.9	9.2	6.7
Non‐Hispanic Mixed Race	5.6	2.8	3.9	2.0	2.0	2.4	1.8	1.0
Non‐Hispanic White	72.4	66.2	70.3	71.9	74.9	77.2	74.0	81.5
*Marital status*:								
Married	51.3	65.4	46.8	64.0	54.1	60.9	33.8	40.8
Divorced, widowed, separated	33.4	23.7	45.3	29.4	43.0	34.5	63.6	56.6
Never married	15.3	10.9	7.9	6.6	3.3	4.6	2.5	2.7
*Education*:								
No degree	21.4	9.0	24.7	7.0	30.4	9.5	32.3	14.8
High school diploma	61.3	45.0	54.3	46.8	50.2	45.1	52.3	51.3
Some college or beyond	17.3	46.0	21.0	46.2	19.4	45.5	15.4	33.9
*Born in United States*	90.9	81.0	91.0	85.0	87.4	86.3	82.9	88.7
*Family income as % of poverty line*:								
Poor/negative	31.5	8.1	17.1	8.2	10.5	7.5	14.0	11.7
Near poor	5.4	2.9	8.2	3.8	6.6	5.5	7.3	4.4
Low income	15.7	8.8	16.2	9.9	23.7	12.5	31.9	19.8
Middle income	20.4	25.6	28.4	25.3	37.0	27.5	27.3	28.4
High income	27.0	54.6	30.2	52.8	22.2	47.0	19.5	35.6
*Insurance coverage*:								
Private	43.7	80.4	50.2	71.3	36.4	56.0	40.6	53.9
Public	46.0	11.7	45.1	25.6	63.7	43.7	59.2	45.9
Uninsured	10.4	7.9	4.7	3.2	0.0	0.3	0.02	0.02
*Dental insurance*	23.1	56.2	21.6	38.6	11.5	18.0	5.9	12.2
*Active smoker within past 12 months*	28.0	8.8	25.4	7.5	10.8	4.8	3.3	0.6

Table 3 shows that the association between age and edentulism increased with age. Relative to people aged 50–59 years, older adults aged 60–69 years of age had 1.65 the odds of edentulism (95% confidence interval: 1.33–2.05) and those aged ≥80 years had 5.84 the odds of edentulism (95% confidence interval: 4.63–7.36). Odds ratios adjusted for sex, race/ethnicity, and marital status were similar to the crude odds ratios suggesting that age related increases in edentulism were not explained by differences in these factors. Additional adjustment for income, smoking status, and dental insurance resulted in slightly attenuated odds ratios. For example, relative to people aged 50–59 years, older adults aged 60–69 years of age had 1.58 the odds of edentulism (95% confidence interval: 1.27–1.96) and those aged ≥80 years had 4.96 the odds of edentulism (95% confidence interval: 3.90–6.31).

**TABLE 3:** Association between age and edentulism (2017)

Age (years)	Percent with edentulism	Crude		Partially adjusted^a^		Fully adjusted^b^	
		Odds ratio	95% Confidence interval	Odds ratio	95% Confidence interval	Odds ratio	95% Confidence interval
50–59	6.2	Reference group					
60–69	9.8	1.65	1.33–2.05	1.67	1.36–2.08	1.58	1.27–1.96
70–79	15.6	2.81	2.28–3.48	2.89	2.34–3.57	2.53	2.05–3.12
≥80	27.7	5.84	4.63–7.36	5.73	4.54–7.24	4.96	3.90–6.31

a Adjusted for sex, race/ethnicity, and marital status.

b Adjusted for variables included in the partially adjusted model *and* family income, smoking status, and dental insurance.

Figure 2 shows the prevalence of oral health care provider visit within 12 months by age among those with and without edentulism. For persons with edentulism, as age increases the prevalence of an oral health care provider visit decreased, whereas for those without edentulism, the prevalence appeared to increase. Table 4 shows that among those with edentulism, the prevalence of an oral health care provider visit in the last 12 months was 23.7% among older adults aged 50–59 years of age, which steadily declined such that the prevalence among those ≥80 years of age was 11.9%. Crude, partially adjusted, and fully adjusted models yielded similar results. After adjusting for sex, race/ethnicity, marital status, income and dental insurance, the association between age and decreased prevalence of oral health care provider visits remained (fully adjusted odds ratio 60–69 years: 0.58; 95% confidence interval: 0.36–0.95; fully adjusted odds ratio 70–79 years: 0.51; 95% confidence interval: 0.30–0.88; ≥ 80 years: 0.45; 95% confidence interval: 0.26–0.80). Among those without edentulism, estimates of oral health visits were 47.4% among those aged 50–59 years, 54.2% among those 60–69 years of age, 59.3% among those 70–79 years of age and 51.0% among those ≥80 years of age. After adjusting for sex, race/ethnicity, marital status, income and dental insurance, adults aged 60–69 years (fully adjusted odds ratio: 1.49; 95% confidence interval: 1.28–1.73), aged 70–79 years (fully adjusted odds ratio: 2.11; 95% confidence interval: 1.81–2.47), aged ≥80 years (fully adjusted odds ratio: 1.69; 95% confidence interval: 1.36–2.10) had increased odds of oral health care provider visits than those aged 50–59 years.

**TABLE 4:** Association between age and oral health care provider visit in the last 12 months, stratified by edentulism (2017)

Age (years)	Percent who visited an oral health care provider	Crude		Partially adjusted^a^		Fully adjusted^b^	
		Odds ratio	95% Confidence interval	Odds ratio	95% Confidence interval	Odds ratio	95% Confidence interval
Among those with edentulism (weighted n = 12,758,419)							
50–59	23.7	Reference group					
60–69	16.0	0.61	0.38–0.99	0.61	0.38–0.98	0.58	0.36–0.95
70–79	13.0	0.48	0.28–0.81	0.50	0.29–0.84	0.51	0.30–0.88
≥ 80	11.9	0.43	0.25–0.77	0.43	0.24–0.75	0.45	0.26–0.80
Among those without edentulism (weighted n = 99,354,222)							
50–59	47.4	Reference group					
60–69	54.2	1.31	1.15–1.49	1.30	1.14–1.48	1.49	1.28–1.73
70–79	59.3	1.61	1.39–1.87	1.60	1.38–1.85	2.11	1.81–2.47
≥ 80	51.0	1.15	0.94–1.41	1.18	0.97–1.45	1.69	1.36–2.10

a Adjusted for sex, race/ethnicity, and marital status.

b Adjusted for variables included in the partially adjusted model *and* family income and dental insurance.

## 5 DISCUSSION

There were two main findings from our study. First, using population‐based contemporary data, this study confirms the association between advanced age and edentulism. Overall 11.4% of adults aged ≥50 years were edentulous; the prevalence increased in those with advanced age. While 6.2% of those aged 50–59 years were edentulous, 27.7% of those ≥80 years of age were edentulous. Second, adherence to guidelines regarding annual oral health provider visits was low with about half of those without edentulism and one in six of those with edentulism reporting a visit with an oral health provider in the past year. Furthermore, the relationship between age and use of oral health services in the past 12 months differed by edentulism status. Adherence to annual oral health care visits was less prevalent in older age groups among edentulous adults and was more prevalent in older age groups among non‐edentulous adults.

Using contemporary data, our population‐based study confirmed the association between advanced age and edentulism. While there is some debate about the factors that contribute to complete tooth loss, people are more likely to lose their natural teeth as they age (Griffin et al., 2012; Hybels et al., 2016; Kanasi et al., 2016). America faces “a silent epidemic” of oral diseases and older adults are at greatest risk (Centers for Disease Control, n.d.). In the United States, older adults develop coronal caries at “approximately one new cavity per year” (Griffin, Griffin, Swann, & Zlobin, 2004; Griffin, Griffin, Swann, & Zlobin, 2005). Despite the rapidly growing older adult population, no recent national data exist for adults aged ≥75 years. It is prudent to understand the oral health needs of aging populations given the United States, and global demographic changes (US Census Bureau: American Fact Finder, n.d.; Harford, 2009). Emerging research indicates a decline in edentulism in some European nations, which varies by country and health policy (Mueller, Naharro, & Carlsson, 2007). Notably, a recent study of community‐dwelling persons ages ≥65 in Italy found a 44% prevalence of edentulism among participants with some 17.5% of persons with edentulism using no protheses (Musacchio et al., 2007). Further research is needed to examine the potential financial expenditures of caring for aging persons oral health needs (Harford, 2009) as well as determining the availability of a qualified workforce.

Adherence to recommendations for annual oral health visits is poor among older adults. As such, population‐level analyses examining the use of oral health services by age are important given the oral health care needs for this vulnerable population (Griffin et al., 2012). The oldest edentulous people in need of routine care are the least likely to receive it. Medicare does not offer routine oral health services as part of the basic health coverage (The Official U.S. Government Medicare Handbook, n.d.). Individuals aged 60–69 years are likely to retire and may have to purchase additional coverage from Medicare, which could be impacting the oral health conditions of this age group. Medicare Advantage plans often include supplemental dental insurance. In 2016, 41% of beneficiaries had supplemental dental insurance (Willink, 2019). That adherence to annual oral health care visits remains suboptimal suggests that additional barriers may prevent older adults from adhering to guidelines recommending annual visits, regardless of edentulism status. This warrants further investigation.

Edentulous persons require annual routine care from oral health providers (Felton et al., 2011). Individuals with edentulism require a complete denture to have a fully functional maceration capacity (Ekelund, 1989). Dentures require maintenance, like any device, and oral health providers recommend annual visits to check the fit of the prostheses, and to check the soft and hard tissues of the mouth which changes over time (American Dental Association Denture Care and Maintenance, n.d.). Persons who have ill‐fitting dentures are at four times higher risk for head and neck cancer, in addition to other health risks (American College of Prosthodontists: Position Statement, n.d.). Only 16% of the overall 13 million persons who have edentulism reported visiting an oral health care provider in the last 12 months. That number in itself is troubling given the maintenance required for a complete denture. Unfortunately, the likelihood of a person visiting an oral health provider decreases with age, leaving persons who are more likely to have edentulism being the least likely to visit an oral health care provider. In our study, adjusting for dental insurance did not explain the decline in adherence to recommended annual oral health care visits. As such, lack of dental insurance may not be the rate limiting factor. Further research to understand factors associated with lack of adherence to routine oral health provider care among older adults is warranted.

The study strengths and limitations must be considered. Data were drawn from a nationally representative sample that provides vital insight into the oral health status of aging persons in the United States, and oral health utilization of a vulnerable group of persons (Christian et al., 2013). Our primary outcome variables from MEPS household data are self‐reported and susceptible to response bias. People may feel uncomfortable speaking about their oral health and concerned about social perceptions if they have edentulism (Lee, Shieh, Yang, Tsai, & Wang, 2007). MEPS interviews are conducted over the phone and persons are able to respond to the interviewers without fear of visual feedback (Hill et al., 2011). Further, studies support the validity of self‐reported dentition in older adults (Douglass, Berlin, & Tennstedt, 1991).

## 6 CONCLUSION

Edentulism is affecting a significant portion of our non‐institutionalized persons aged ≥50 years and has a profound impact on diet, overall health, and pre‐existing conditions (Polzer, Schimmel, Mueller, & Biffar, 2011). People need teeth in order to chew and they require functional, well‐cared for prostheses if they do not have a natural dentition. Our data show that persons are not receiving the annual care required to care for their complete denture, and that lack of dental insurance does not explain the age‐related decrease in prevalence of adherence to annual oral health care provider visits. Research is needed to understand how to better improve adherence to recommended annual oral health care provider visits for aging populations, particularly among older edentulous adults who have the greatest need for intervention.

## AUTHOR CONTRIBUTIONS

Andriana M. Foiles Sifuentes and Kate L. Lapane and Maira A. Castaneda‐Avila made substantial contributions to the conception, design, analysis, and interpretation of study findings. Andriana M. Foiles Sifuentes wrote the original draft of the article and Kate L. Lapane and Maira A. Castaneda‐Avila critically revised the manuscript, providing important intellectual content. All authors approved the final version of the paper.
